# Proline Accumulation in Pollen Grains as Potential Target for Improved Yield Stability Under Salt Stress

**DOI:** 10.3389/fpls.2020.582877

**Published:** 2020-10-28

**Authors:** Roberto Mattioli, Noemi Palombi, Dietmar Funck, Maurizio Trovato

**Affiliations:** ^1^Department of Biology and Biotechnology, Sapienza University of Rome, Rome, Italy; ^2^Department of Science, Roma Tre University, Rome, Italy; ^3^Department of Plant Physiology and Biochemistry, University of Konstanz, Konstanz, Germany

**Keywords:** proline, pollen, seed yield, salt stress, *P5CS2*, stress tolerance

## Abstract

Seed yield, a major determinant for the commercial success of grain crops, critically depends on pollen viability, which is dramatically reduced by environmental stresses, such as drought, salinity, and extreme temperatures. Salinity, in particular, is a major problem for crop yield known to affect about 20% of all arable land and cause huge economic losses worldwide. Flowering plants are particularly sensitive to environmental stress during sexual reproduction, and even a short exposure to stressing conditions can severely hamper reproductive success, and thus reduce crop yield. Since proline is required for pollen fertility and accumulates in plant tissues in response to different abiotic stresses, a role of proline in pollen protection under salt stress conditions can be envisaged. In this perspective, we analyze old and new data to evaluate the importance of pollen development under saline conditions, and discuss the possibility of raising proline levels in pollen grains as a biotechnological strategy to stabilize seed yield in the presence of salt stress. The overall data confirm that proline is necessary to preserve pollen fertility and limit seed loss under stressful conditions. However, at present, we have not enough data to conclude whether or not raising proline over wildtype levels in pollen grains can effectively ameliorate seed yield under saline conditions, and further work is still required.

## Introduction

Abiotic stresses like extreme temperatures, drought, and high salinity are responsible for strong losses in crop production worldwide and are expected to worsen in the future because of the ongoing climate change (IPPC, 2019). High soil salinity inhibits plant growth and productivity mainly by limiting water uptake, promoting the intracellular accumulation of Na^+^ and Cl^–^, which in turn cause the accumulation of reactive oxygen species (ROS; Rengasamy, 2006; Munns and Tester, 2008; van Zelm et al., 2020). Numerous studies have addressed stress tolerance in plants mainly focusing on germination or the vegetative phase with comparatively little attention paid to the reproductive stage. In flowering plants, however, the reproductive phase is highly sensitive to abiotic stresses (Dolferus et al., 2011; Senapati et al., 2019).

## Pollen Fertility and Stress Tolerance

Most of the yield reduction caused by heat and drought stress are caused by hampered pollen viability and reduced ovule fertilization (Barnabas et al., 2008). The sensitivity of the reproductive phase to drought is especially evident in rice, where water stress caused incomplete anther and pollen development, incomplete anther dehiscence, a strong reduction in grain set, and precocious grain abortion soon after fertilization (Namuco and O’Toole, 1986; Ekanayake et al., 1989; Garrity and O’Toole, 1994; Guo et al., 2016). A positive relationship between grain yield and pollen fertility under drought or heat conditions also has been described in wheat (Lalonde et al., 1997; Yu et al., 2019). However, ovule and embryo development can also be involved in stress-induced sterility and reduced seed setting of grain crops (Wilkinson et al., 2018).

Salt stress experienced during the reproductive phase dramatically impedes pollen development and viability, as shown by *in vitro* pollen viability and germination tests from Petunia, maize, and carrot plants grown under saline conditions (Reddy and Goss, 1971; Dhingra and Varghese, 1985; El Sayed and Kirkwood, 1992; Kiełkowska et al., 2019). Reduced seed set as a consequence of hampered pollen fertility was also reported in rice (Abdullah et al., 2001; Gerona et al., 2019) and wheat (Abdullah et al., 1978) grown in saline soils. Direct experimental evidence for a decisive role of pollen quality in maintaining fertility and yield under salinity conditions was obtained in barley: The lower yield of a salt-sensitive cultivar was overcome by cross-pollination with pollen from a salt-tolerant cultivar (Hirasawa et al., 2017; Kodama et al., 2018).

## Proline and Stress Tolerance

Although stress tolerance in plants is a complex process brought about by the coordinated action of different mechanisms (Munns and Gilliham, 2015), the accumulation of single compatible solutes has been reported to confer stress tolerance in several plant species (Biancucci et al., 2015; Hossain et al., 2019). Proline has long been known to accumulate upon abiotic and, to a lesser extent, biotic stresses, and the accumulation of this amino acid has been frequently correlated with improved stress tolerance (Forlani et al., 2019). More recently, transcriptomic and proteomic studies (Gupta et al., 2016; Hoermiller et al., 2017; Batista-Silva et al., 2019), genetic analyses (Xia et al., 2017) and meta-analyses of publicly available datasets (Hildebrandt, 2018; Guo et al., 2019), have corroborated the importance of proline metabolism in stress tolerance.

**FIGURE 1:**
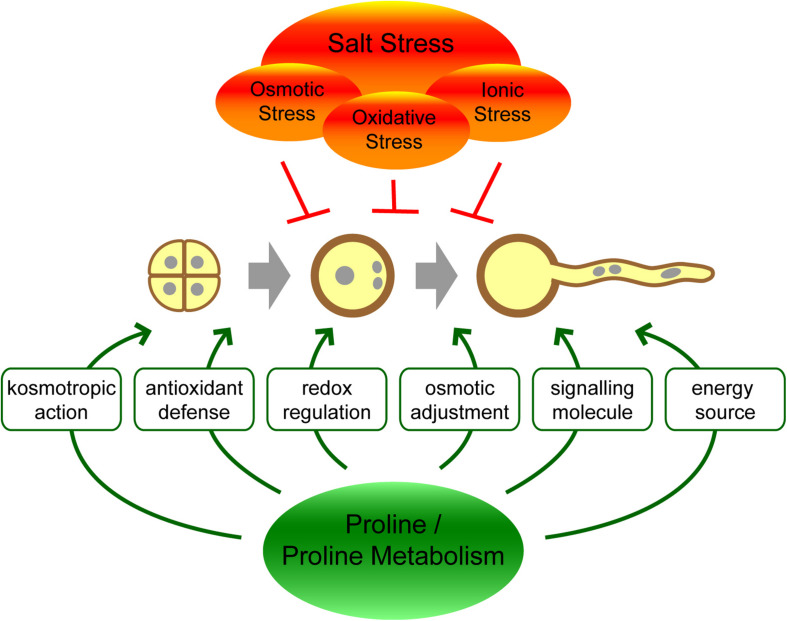
Hypothetical functions of proline or proline metabolism in the protection of pollen against salt stress.

The molecular mechanisms by which proline exerts its effects on stress tolerance are not fully understood, and different functions may act together or in parallel (Figure 1). As a highly soluble zwitterionic molecule, proline can accumulate to high concentrations in plant cells and contribute to the osmotic potential. In this way, proline could limit the loss of water from cells during drought or salt stress conditions. While proline accumulation can significantly contribute to the osmotic pressure in halophytes, the biological significance of this mechanism has been questioned in glycophytes and might be only marginal (Munns and Tester, 2008; Forlani et al., 2019). With its kosmotropic properties, proline can facilitate the correct folding of proteins or counteract protein denaturation caused by water stress (Kumar, 2009; Forlani et al., 2019; Mojtabavi et al., 2019). Additionally, proline can prevent protein aggregation at low water availability or high ionic strength by modulating surface charges and Coulomb interactions (Govrin et al., 2018; Rydeen et al., 2018; Govrin et al., 2019). Alternatively or in addition, proline synthesis might improve stress tolerance in plants by modulating the NADP^+^/NADPH ratio in the cytosol, resulting in crosstalk with other stress-related pathways like the oxidative pentose phosphate pathway (Sharma et al., 2011; Esposito, 2016). Last but not least, proline might also behave as a second messenger participating in signal transduction pathways leading to stress tolerance (Hayat et al., 2012; Liang et al., 2013).

A secondary effect of salinity, following osmotic and ionic stress, is oxidative stress, which leads to accumulation of reactive oxygen species (ROS), the removal of which represents an important mechanism of stress tolerance. Although a particular action of proline as ROS scavenger is unlikely (Signorelli et al., 2014), proline has been reported to reduce the accumulation of hydrogen peroxide, limit the peroxidation of cell lipids, and enhance the activities of antioxidant enzymes (Banu et al., 2009; Ben Rejeb et al., 2014, 2015; Guo et al., 2020; Hanif et al., 2020; Liu et al., 2020). Both the stabilization of antioxidant enzymes and the induction of gene expression have been suggested. A direct sensor for proline has so far not been identified in plants, but proline catabolism in mitochondria was shown to increase the production of ROS, which may in turn act to induce antioxidant enzyme expression (Liang et al., 2013; Ben Rejeb et al., 2014). Interestingly, proline degradation was also shown to induce the activity of plasma membrane NADPH oxidases, which in turn stimulates proline biosynthesis, potentially generating a feed-forward loop (Ben Rejeb et al., 2015; Fabro et al., 2016).

Intriguingly, proline also accumulates under non-stressed conditions after floral transition, mainly in reproductive tissues and organs, and is thought to contribute to the regulation and success of reproduction. The role of proline in plant sexual reproduction seems particularly important for pollen grains, where it accumulates in large amounts and is essential for pollen development and fertility (Trovato et al., 2019). Proline accumulation is mainly achieved by synthesis from glutamate through the sequential action of δ^1^-pyrroline-carboxylate synthetase (P5CS) and δ^1^-pyrroline-carboxylate reductase (P5CR). Like many seed crops, the model plant Arabidopsis contains two P5CS isoforms coded for by *P5CS1*, and *P5CS2*, respectively, while P5CR is encoded by a single *P5CR* gene (Turchetto-Zolet et al., 2009; Funck et al., 2012; Rai and Penna, 2013). Mitochondrial degradation mediated by proline dehydrogenase (ProDH) and δ^1^-pyrroline-carboxylate dehydrogenase (P5CDH/GSALDH) can limit proline accumulation (Trovato et al., 2019). ProDH is also present in two isoforms in Arabidopsis, encoded by *ProDH1* and *ProDH2*, whereas P5CDH, which is also required for arginine degradation, is encoded by a single copy gene (Deuschle et al., 2001; Funck et al., 2010).

Despite the rapid and massive increase of proline after water stress, the causal connection between proline accumulation and stress tolerance remains a matter of debate. Although an overwhelming number of reports claim that proline accumulation leads to stress tolerance (Forlani et al., 2019), some authors have raised concerns hypothesizing that proline metabolism, rather than proline accumulation, might be responsible for stress tolerance (Bhaskara et al., 2015), or have challenged the notion that proline confers stress tolerance (Kim et al., 2016; Mwadzingeni et al., 2016; Mansour and Ali, 2017; Forlani et al., 2019). Signorelli (2016) proposed that the accumulation of proline during stress could be a mere consequence of the metabolic adaptation of plant cells to stress. Toxic effects of external proline application suggested that high proline content is not *per se* beneficial for plants under all conditions (Hellmann et al., 2000).

## Proline and Stress Tolerance During Seed Production

Some lines of evidence, however, may suggest a special role for proline in protecting reproductive processes under stressful conditions. In a study from Mwadzingeni et al. (2016) on ninety-six bread wheat (*Triticum aestivum* L.) genotypes, conducted under both greenhouse and field conditions, a weak but positive correlation between grain yield and proline content in vegetative tissue was detected. Strikingly, the beneficial role for proline accumulation in maintaining productivity was only evident when the drought stress was applied during flowering and grain-filling periods. Essential or beneficial roles of high proline contents were reported in several aspects of plant reproduction, such as flowering time, embryogenesis, and pollen development and fertility (Székely et al., 2008; Trovato et al., 2019). Also exogenous proline has been reported to stimulate yield under salt stress, although the mechanisms by which external proline treatment can mitigate the negative effects of salt remain elusive and may not be identical to those of endogenous proline accumulation (Farooq et al., 2017; Meena et al., 2019; El Moukhtari et al., 2020). In wheat grown in the presence of 120 mM NaCl, for example, pretreatment with proline increased fresh and dry biomass, grain yield and grain weight (Rady et al., 2019). In maize treated with different concentrations of NaCl, proline solutions sprayed on maize leaves improved growth and grain yield at 25 mM and 50 mM NaCl (Alam et al., 2016). To the best of our knowledge, the economic feasibility of using external proline application as an agricultural practice has never been analyzed.

The increased abundance of proline observed in reproductive tissues after floral transition in Arabidopsis (Chiang and Dandekar, 1995) may reflect the specific need for stress protection during the reproductive phase. In support of this hypothesis, Sulpice et al. (2003) reported that treatment of Arabidopsis plants with 100 mM NaCl caused both pollen and ovule sterility, leading to a strong reduction of the number of seeds per silique. These defects were partially rescued in transgenic Arabidopsis engineered to accumulate glycine betaine, a non-proteinogenic amino acid with similar properties as proline (Sulpice et al., 2003; Waditee et al., 2005).

**FIGURE 2:**
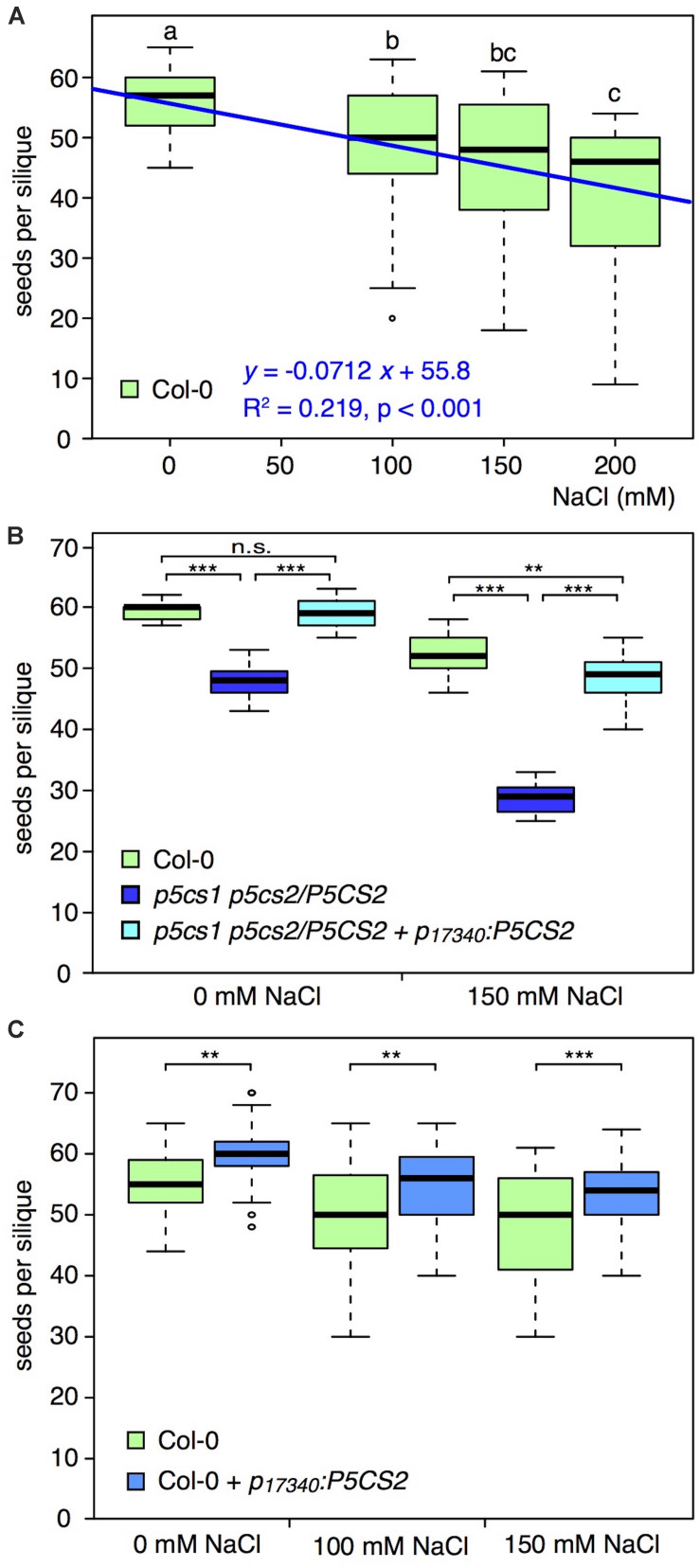
Effects of salt on the number of seeds per silique in different genotypes. **(A)** Box plot and regression line (blue) showing the effects of different NaCl concentrations on the number of seeds per silique in wildtype (Col-0) plants. Different letters indicate significant differences (*p* < 0.05, *n* = 31 siliques per salt concentration) between groups calculated by all-pairwise Mann-Whitney-Wilcoxon tests. **(B)** Box plot of the numbers of seeds per silique in wildtype (green), *p5cs1 p5cs2/P5CS2* sesquimutants (dark blue), and *p5cs1 p5cs2/P5CS2* + *p_17340_:P5CS2* (light blue), treated without or with 150 mM NaCl. Asterisks indicate significant differences between genotypes (**: *p* < 0.01, ***: *p* < 0.001, n.s.: not significant; *n* = 15 siliques per genotype and condition). All genotypes had significantly reduced numbers of seeds per silique under salt stress and there was a significant interaction between salt stress and genotype. **(C)** Box plot showing the numbers of seeds per silique in wildtype (green) and *p_17340_:P5CS2* (blue) treated with 0, 100, and 150 mM NaCl. Asterisks indicate significant differences between genotypes (**: *p* < 0.01, ***: *p* < 0.001; 28 < n < 74 siliques per genotype and condition). Both genotypes showed significantly reduced numbers of seeds per silique in response to salt stress but there was no significant interaction between salt stress and genotype. In all the panels, boxes indicate the lower and upper quartiles, and the solid horizontal line shows the median. Whiskers above and below the box display the 90th and 10th percentile, respectively, and outliers are shown as open circles. Representative data sets from at least three replicates with independent transgenic lines are shown. For details about statistical analyses please refer to Supplementary Tables 1–3.

To verify a possible correlation between grain yield and proline content, we carried out a series of experiments with the proline deficient sesquimutant *p5cs1 p5cs2/P5CS2* (homozygous for a *p5cs1* mutation and heterozygous for *p5cs2*), and analyzed the number of seeds per silique under moderate salt stress conditions (For experimental details see Supplementary Methods S1). Similar to Sulpice et al. (2003), we found that salt stress treatment, administered after floral transition, led to a significant and proportional reduction in the number of seeds per silique of wildtype plants (Figure 2A and Supplementary Table S2). When we exposed the *p5cs1 p5cs2/P5CS2* mutant to 150 mM NaCl, the detrimental effect was even more pronounced than in wildtype plants, and we recorded significantly stronger reduction in the number of seeds per silique (Figure 2B and Supplementary Table S3), supporting, in the converse argument, the hypothesis that proline may reduce salinity damages on seed production. While these data confirm the importance of proline for seed set under salt stress conditions, they do not clarify whether or not it is specifically important for pollen protection, because all tissues of a *p5cs1 p5cs2/P5CS2* mutant are poor in proline. The content of free proline measured in *p5cs1 p5cs2/P5CS2* mutants varies depending on different tissues and growth conditions, but, on average, these sesquimutants have from one-third to one-fourth as much proline as wildtype plants (Funck et al., 2012; Mattioli et al., 2012, 2018).

Data from *p5cs1 p5cs2/P5CS2 p_17340_:P5CS2*, a *p5cs1 p5cs2/P5CS2* mutant expressing an additional copy of the proline biosynthesis gene *P5CS2* under the control of the pollen-specific promoter of the *At5g17340* gene, encoding a putative membrane lipoprotein (Honys and Twell, 2004; Mattioli et al., 2018), though, provided further support for the hypothesis that proline is especially important in pollen protection under stress. The number of seeds per silique in a *p5cs1 p5cs2/P5CS2 p_17340_:P5CS2* line was significantly higher compared to the parental *p5cs1 p5cs2/P5CS2* sesquimutants under stress conditions, and close to wildtype numbers (Figure 2B and Supplementary Table S2). Since the pollen-specific expression of *p5cs1 p5cs2/P5CS2 p_17340_:P5CS2* can complement the defect of proline synthesis in pollen grains but not in other tissues (Mattioli et al., 2018), the fewer seeds found in sesquimutant siliques are likely caused by insufficient proline in pollen grains. At present, however, we cannot rule out a possible contribution of ovule fertility in the overall process of seed production under the tested conditions, and further investigations are clearly needed.

## Does Enhanced Proline Accumulation in Pollen Grains Improve Seed Production Under Salt Stress?

Despite the encouraging indications coming from the literature, and from our experiments with mutants defective in proline synthesis, clear-cut evidence that enhanced proline accumulation in pollen grains can sustain seed production under stress is still lacking. In an attempt to clarify this point, we introduced the pollen-specific *p_17340_:P5CS2* construct in wildtype Arabidopsis plants and analyzed the resulting transgenic plants for the number of seeds per silique. In preliminary results from heterozygous T1 progeny directly after the selection, most transgenic lines produced more seeds per silique. In the T2 generation, we found several homozygous lines bearing significantly more seeds per silique than wildtype, while others produced similar or even lower seed numbers. However, when we exposed the plants with more seeds per silique to 100 or 150 mM NaCl after floral transition, we observed a decline in seed numbers both in wildtype and in *p_17340_:P5CS2* transgenic plants (Figure 2C and Supplementary Table S4), with similar trendline slopes of −0.046 for wildtype and −0.044 for *p_17340_:P5CS2.* At 200 mM NaCl, the seed numbers per silique were on average lower in the *p_17340_:P5CS2* transgenic plants but the data were too variable for both genotypes and could not be meaningfully included in the statistical analysis (data not shown).

At this stage, we can say that expression of an additional copy of *P5CS2* in pollen during late stages of development, as mediated by the *At5g17340* promoter, improves pollination and fertilization success. However, we cannot draw a final conclusion whether enhanced proline accumulation in pollen could improve the yield stability under salt stress conditions.

Although this result may corroborate the hypothesis that proline accumulation is not causal for salt stress tolerance, there are still good reasons for further investigations. A possible reason for the unchanged salt sensitivity of our *p_17340_:P5CS2* transgenic plants could be that we chose a wrong pollen-specific promoter. Although very effective in a proline-deficient mutant, this promoter might not be strong enough to provide a significant proline surplus in a wildtype background subjected to salt stress, when the expression of the endogenous *P5CS1* gene is already induced (Strizhov et al., 1997). Alternatively, the protective capacity of proline accumulation or biosynthesis could already be exhausted in pollen and any further increase may draw resources from other protective mechanisms.

## Future Perspectives

Proline accumulation, especially in pollen, has undoubtedly a major role in the critical phase of seed development under stress, as it has been shown earlier and was reinforced in this work. At present, direct experimental proof is still lacking that it is possible to raise proline levels in pollen grains above physiological levels, and that such an increase could effectively improve seed yield under (salt) stress conditions. The *At5g17340* promoter used in this study is expressed in gametophytic, but not in sporophytic tissues of the anther during late phases of pollen maturation (da Costa-Nunes, 2013; Mattioli et al., 2018). It is possible that, under salt stress conditions, increased proline content or biosynthesis would additionally be required during meiosis and early microspore development or in sporophytic tissues, particularly the tapetum, to improve pollen development and quality. Alternative or artificially enhanced promoters may be used to boost P5CS expression in the male reproductive tissue.

Feedback regulation of both proline biosynthesis and degradation have been demonstrated and may prevent or limit the further increase in proline levels in pollen. In particular, the feedback inhibition exerted by proline on the activity of P5CS1 and P5CS2 (Zhang et al., 1995) can potentially limit proline accumulation in pollen grains and might be removed by mutagenesis of a critical, conserved phenylalanine residue (Zhang et al., 1995; Hong et al., 2000), through gene editing. Both, the expression of *ProDH1* and, to a lesser extent, *ProDH2* were shown to be upregulated by proline in Arabidopsis via the transcription factor bZIP11 (Satoh et al., 2004; Hanson et al., 2008). The binding sites of bZIP11 in the promoters of *ProDH1* and *ProDH2* are known and could be modified or blocked by an artificial binding protein. Alternatively, *DFR1*, encoding a mitochondrial protein potentially inhibiting proline degradation (Ren et al., 2018) might be overexpressed in pollen. In addition, it is known that different plant species, and even different natural mutants of a same species, can use different post-transcriptional, post-translational and epigenetic regulatory mechanisms to adjust proline homeostasis, including alternative splicing (Kesari et al., 2012), ubiquitination (Ju et al., 2013; Kim et al., 2017), histone trimethylation (Feng et al., 2016) and microRNA-mediated regulation (Gupta, 2020).

To improve the chances of successfully modifying stress tolerance of pollen development, a better knowledge of the metabolites and metabolic processes important for microspore development will be required. In a recent work, Wada et al. (2020) used on-site cell-specific mass spectrometry to analyze the metabolome of single pollen grains under normal and heat stress conditions, and found proline as one of the most abundant metabolites. This new technology might be used to identify mutants with increased proline content and to determine if proline accumulation is at some point limited by precursor availability or may interfere with other metabolic pathways. In parallel, the recent improvements of single-cell transcriptomics will facilitate the comparison between different developmental phases of pollen in the absence or presence of stress, and may help to identify additional genes that are essential for pollen viability under stress. All experiments that aim to enhance the stress tolerance of pollen should be carried out and verified in different stress conditions and carefully designed, not only *in vitro* but also in field conditions, to evaluate in real conditions the feasibility of this strategy to improve crop resilience to salinity stress.

## Data Availability Statement

The original contributions presented in the study are included in the article/Supplementary Material, further inquiries can be directed to the corresponding author.

## Author Contributions

MT and DF organized and wrote the manuscript and performed the statistical analyses. RM generated the transgenic plants. NP performed the stress experiments and the count of seeds. All authors have read and approved the manuscript.

## Conflict of Interest

The authors declare that the research was conducted in the absence of any commercial or financial relationships that could be construed as a potential conflict of interest.
