# Supplementary material for: Proline Accumulation in Pollen Grains as Potential Target for Improved Yield Stability Under Salt Stress
Source: Front Plant Sci. 2020 Oct 28;11:582877. doi: 10.3389/fpls.2020.582877 (PMC7655902; doi:10.3389/fpls.2020.582877)
Supplement: Supplementary file 1 [file Data_Sheet_1.PDF]

Supplementary Materials S1

Growth conditions and salt stress treatment

Wildtype and mutant *Arabidopsis thaliana* (Col-0) were grown in a growth chamber at 24/21°C with a light intensity of 300-µE·m-2·s-1, and a light/night cycle of 16/8 hours per day. Stress experiments were performed after anthesis, by watering plants twice a week with a saline solution for 20 days. To ensure that plants with different flowering time, or unsynchronized flowering, were given the same stress treatment, we grew plants in individual pots and moved them to experimental trays after floral transition, marking the first inflorescence with a ribbon.

Seed analysis

For seed analysis, siliques were dissected and analyzed under a Zeiss Stevi SV 6 light stereo-microscope (Carl Zeiss Microimaging GmbH, Jena, Germany). Digital images were acquired with a Jenoptik ProgResW C3 digital camera (Jenoptik, Jena, Germany), and seeds were counted with the aid of the free open source ImageJ 1.x program (Schneider et al., 2012). All the analyses have been repeated at least four times.

Generation of transgenic plants

The *p5cs1 p5cs2/P5CS2* p_{17340}*:P5CS2* (expressing an additional copy of *P5CS2* under control of the pollen-specific promoter of *At5g17340*) mutant used in this work was described in (Mattioli et al., 2018). Since the homozygous *p5cs2* is embryo lethal (Székely et al., 2008; Mattioli et al., 2009), all the *p5cs1 p5cs2/P5CS2* p_{17340}*:P5CS2* mutants used in the analyses were selected at germination with 12 µg/ml Sulfadiazine to select heterozygous *p5cs2/P5CS2*. Transgenic homozygous p_{17340}*:P5CS2* plants, containing a p_{17340}*:P5CS2* in a Col-0 background, were generated by introducing *p17340:P5CS2* in a Col-0 background via floral dipping (Clough and Bent, 1998) or backcrossing. Kanamycin selection was used to isolate T1 transformants, T2 single-insertion lines, and T3 homozygous lines. Presence of the desired transgene in each line was confirmed by PCR using the primers 5-agggagcatgcataagatatcga and 5- tcgaagcctttgtcgaccaa. PCR conditions were 3' at 94°C followed by 35 cycles of 30" at 94°C, 30" at 59°C, and 30" at 72°C. Twenty independent single-insertion lines were isolated, from which three homozygous lines were selected and used for subsequent investigations.

Statistical analysis

Statistical analyses were performed with R version 3.6.3 (R core team, 2019). The data for seeds per silique or the residuals of simple linear models were in many cases not distributed normally, therefore we had to apply different tools for the analysis of main effects and pairwise comparisons to avoid violating model constraints. Details about the analyses and the results can be found in supplementary tables 1-3. All experiments were repeated at least three times with independent homozygous lines.

Bibliography

Clough, S.J., and Bent, A.F. (1998). Floral dip: a simplified method for *Agrobacterium*-mediated transformation of *Arabidopsis thaliana*. *Plant Journal* 16(6)**,** 735-743.

Mattioli, R., Falasca, G., Sabatini, S., Altamura, M.M., Costantino, P., and Trovato, M. (2009). The proline biosynthetic genes *P5CS1* and *P5CS2* play overlapping roles in Arabidopsis flower transition but not in embryo development. *Physiologia Plantarum* 137(1)**,** 72-85.

R core team (2019). "R: A language and environment for statistical computing. R Foundation for Statistical Computing". (vienna, Austria).

Schneider, C.A., Rasband, W.S., and Eliceiri, K.W. (2012). NIH Image to ImageJ: 25 years of image analysis. *Nature methods* 9(7)**,** 671-675.

Székely, G., Ábrahám, E., Cséplő, A., Rigó, G., Zsigmond, L., Csiszár, J., et al. (2008). Duplicated *P5CS* genes of Arabidopsis play distinct roles in stress regulation and developmental control of proline biosynthesis. *Plant Journal* 53(1)**,** 11-28.
