# Supplementary material for: Proline Accumulation in Pollen Grains as Potential Target for Improved Yield Stability Under Salt Stress
Source: Front Plant Sci. 2020 Oct 28;11:582877. doi: 10.3389/fpls.2020.582877 (PMC7655902; doi:10.3389/fpls.2020.582877)
Supplement: Supplementary file 2 [file Data_Sheet_2.PDF]

Supplementary Table S2: Statistical analyses of the influence of different NaCl concentrations on the number of seeds per silique in Col-0 wildtype plants (Figure 1A)

**All-pairwise comparisons ***			
comparison	p-value	comparison	p-value
0 *vs.* 100 mM NaCl	**<0.01**	100 *vs.*150 mM NaCl	0.51
0 *vs.*150 mM NaCl	**<0.001**	100 *vs.*200 mM NaCl	**0.03**
0 *vs.*200 mM NaCl	**<0.001**	150 *vs.*200 mM NaCl	0.09

* using non-paired Mann-Whitney-Wilcoxon rank sum tests with Benjamini & Hochberg's correction for multiple testing. Analysis of the data with a linear model gave very similar results but the model residuals did not pass a normality test.
