# Supplementary material for: Proline Accumulation in Pollen Grains as Potential Target for Improved Yield Stability Under Salt Stress
Source: Front Plant Sci. 2020 Oct 28;11:582877. doi: 10.3389/fpls.2020.582877 (PMC7655902; doi:10.3389/fpls.2020.582877)
Supplement: Supplementary file 3 [file Data_Sheet_3.PDF]

Supplementary Table S3: Statistical analysis of seed numbers per silique in Col-0, *p5cs1 p5cs2/P5CS2 and p5cs1 p5cs2/P5CS2* + *p_{17340}:P5CS2* under control conditions and stressed with 150 mM NaCl (Figure 1B)

**2-way ANOVA of linear model**					
**Source of Variation**	**Degrees of freedom**	**Sum of squares**	**Mean squares**	**F-value**	**p-value**
Treatment	1	3373	3373	380	**<0.001**
Genotype	2	5329	2664	300	**<0.001**
Treatment x genotype	2	546	273	30.8	**<0.001**
Residual	84	745	8.9		
Shapiro-Wilk test (normal distribution)					0.89
Levene test (homogeneity of variance)	5			2.22	0.06

**Pairwise comparisons** [a]				
**Fixed Factor**	**Comparison**	**Diff of Means**	**t-value**	**p-value**
ctrl	Col-0 *vs.* *p5cs1 p5cs2/P5CS2*	-11.7	-10.7	**<0.001**
	Col-0 *vs.* *p5cs1 p5cs2/P5CS2* +*p_{17340}:P5CS2*	-1.13	-1.04	0.87
	p5cs1 p5cs2/P5CS2 *vs.* *p5cs1 p5cs2/P5CS2* + *p_{17340}:P5CS2*	10.5	9.68	**<0.001**
NaCl	Col-0 *vs.* *p5cs1 p5cs2/P5CS2*	-23.3	-21.4	**<0.001**
	Col-0 *vs.* *p5cs1 p5cs2/P5CS2* + *p_{17340}:P5CS2*	-4.07	-3.74	**<0.01**
	p5cs1 p5cs2/P5CS2 *vs.* *p5cs1 p5cs2/P5CS2*+ *p_{17340}:P5CS2*	19.2	17.7	**<0.001**
Col-0	ctrl *vs.* NaCl	-7.40	-6.80	**<0.001**
p5cs1 p5cs2/P5CS2		-19.0	-17.4	**<0.001**
p5cs1 p5cs2/P5CS2 + *p_{17340}:P5CS2*		-10.3	-9.50	**<0.001**

[a] with the *glht* function of the R-package "multcomp" (Hothorn, T., Bretz, F., and Westfall, P. (2008). Simultaneous inference in general parametric models. *Biom. J.* 50(3), 346-363. doi: 10.1002/bimj.200810425)
