# Supplementary material for: Proline Accumulation in Pollen Grains as Potential Target for Improved Yield Stability Under Salt Stress
Source: Front Plant Sci. 2020 Oct 28;11:582877. doi: 10.3389/fpls.2020.582877 (PMC7655902; doi:10.3389/fpls.2020.582877)
Supplement: Supplementary file 4 [file Data_Sheet_4.PDF]

frontiers

Supplementary Table S4: Statistical analyses of the influence of different NaCl concentrations on the number of seeds per silique in Col-0 and transgenic plants carrying the *p_{17340}:P5CS2* construct (Figure 1C)

Source of variation [a]	DF [b]	AIC [c]	LRT [d]	p-value
Treatment	2	2478	51.0	**<0.001**
Genotype	1	2471	42.1	**<0.001**
Treatment x genotype	2	2430	0.902	0.63
**Pairwise comparisons [e]**				
**Fixed factor**	**comparison**		**z-value**	**p-value**
0 mM NaCl	Col-0 *vs.* Col-0 + *p_{17340}:P5CS2*		3.69	**<0.01**
100 mM NaCl			3.46	**<0.01**
150 mM NaCl			4.16	**<0.001**
Col-0	0 *vs.* 100 mM NaCl		-3.98	**<0.001**
	0 *vs.* 150 mM NaCl		-4.82	**<0.001**
	100 *vs.* 150 mM NaCl		-1.38	0.67
Col-0 + *p_{17340}:P5CS2*	0 *vs.* 100 mM NaCl		-4.35	**<0.001**
	0 *vs.* 150 mM NaCl		-4.42	**<0.001**
	100 *vs.* 150 mM NaCl		-0.17	1.00

[a] A generalized linear model (R-package "*lme4*"; Bates, D., Mächler, M., Bolker, B., and Walker, S. (2015). Fitting Linear Mixed-Effects Models Using *lme4*. *J. Stat. Softw.* 1(1). doi: 10.18637/jss.v067.i01) assuming Poisson-distribution was used to compare different genotypes among each other as well as their response to the different treatments. Significance of the influence of treatment, genotype and their interaction was determined by excluding these factors successively from the model.

[b] Degrees of freedom

[c] Akaike information criterion

[d] Likelihood ratio test

[e] with the *glht* function of the R-package "*multcomp*" (Hothorn, T., Bretz, F., and Westfall, P. (2008). Simultaneous inference in general parametric models. *Biom. J.* 50(3), 346-363. doi: 10.1002/bimj.200810425)
